# Pongol Methyl Ether Inhibits Akt and Suppresses Cancer Stem Cell Phenotypes in Lung Cancer Cells

**DOI:** 10.3390/ph14111085

**Published:** 2021-10-26

**Authors:** Arnon Silapech, Satapat Racha, Nithikoon Aksorn, Pennapa Lafauy, Sucharat Tungsukruthai, Chanida Vinayanuwattikun, Boonchoo Sritularak, Pithi Chanvorachote

**Affiliations:** 1 Cell-Based Drug and Health Product Development Research Unit, Faculty of Pharmaceutical Sciences, Chulalongkorn University, Bangkok 10330, Thailand; bestwander@outlook.com (A.S.); pennapabifern@gmail.com (P.L.); 2 Interdisciplinary Physiology Program, Graduate School, Chulalongkorn University, Bangkok 10330, Thailand; 3 Interdisciplinary Program in Pharmacology, Graduate School, Chulalongkorn University, Bangkok 10330, Thailand; satapatto@gmail.com; 4 Department of Clinical Pathology, Faculty of Medicine Vajira Hospital, Navamindradhiraj University, Bangkok 10300, Thailand; nithikoon@nmu.ac.th; 5 Division of Health and Applied Sciences, Faculty of Science, Prince of Songkla University, Hat Yai, Songkhla 90110, Thailand; sucharat.tungsukruthai@gmail.com; 6 Division of Medical Oncology, Department of Medicine, Faculty of Medicine, Chulalongkorn University, Bangkok 10330, Thailand; Chanida.Vi@chula.ac.th; 7 Department of Pharmacognosy and Pharmaceutical Botany, Faculty of Pharmaceutical Sciences, Chulalongkorn University, Bangkok 10330, Thailand; boonchoo.sr@chula.ac.th; 8 Department of Pharmacology and Physiology, Faculty of Pharmaceutical Sciences, Chulalongkorn University, Bangkok 10330, Thailand

**Keywords:** pongol methyl ether, cancer stem cell, lung cancer, Akt, CSC-targeting

## Abstract

Cancer stem cells (CSCs) are an important therapeutic target. The therapeutic agents targeting CSCs should lead to improved clinical outcomes. Here we have demonstrated the CSC-suppressing activity of pongol methyl ether (PME), a pure compound from *Millettia erythrocalyx*. Methods: CSC-suppressing effects were evaluated by spheroid formation assay and detection of CSC markers. The related CSC cell signals were evaluated by Western blot, immunofluorescence and molecular docking analysis. Proteins affected by PME treatment were subjected to bioinformatic analysis. Protein–protein interaction (PPI) networks were constructed by the Search Tool for Interactions of Chemicals (STITCH). The Kyoto Encyclopedia of Genes and Genomes (KEGG) mapper were used to confirm the underlying pathways. Results: PME (5–25 µM) significantly suppressed the ability of lung cancer cells to form colonies, grow in an anchorage-independent manner and generate tumour spheroids. PME at 25 µM significantly decreased the CSC markers (CD133 and ALDH1A1) and pluripotent transcription factors (Oct4 and Nanog). Akt, the key upstream signal of CSC control, was significantly decreased by the PME treatment. The molecular docking indicated that PME was bound to Akt-1 with a binding affinity of −9.2 kcal/mol greater than the Akt-1 inhibitor (reference compound; CQW). The STITCH network identified a total of 15 proteins interacted in PPI networks, and Akt-1 was identified as a central protein. The KEGG mapper indicated that the selected CSC markers were mostly involved in the ‘signalling pathways regulating pluripotency of stem cells’ pathway map and Akt, Oct4 and Nanog were the regulatory proteins in the dominant pathway. In addition, PME (10–25 µM) can suppress spheroid formation and reduce CSC-specific marker expression in patient-derived primary lung cancer cells. Conclusions: Our study revealed a novel pharmacological effect and the underlying mechanism of PME that can attenuate CSC phenotypes in lung cancer cells and may be developed for lung cancer therapy.

## 1. Introduction

Lung cancer is a major public health problem worldwide with high incidence and mortality rate [1]. Prominent evidence shows that cancer stem cells (CSCs) in lung cancer contribute to tumour recurrence and failure of therapy [2]. CSCs have special abilities including resisting chemotherapeutic drugs, surviving in detached conditions via the anoikis resistant mechanisms and initiating new tumour at secondary site [2]. CSCs are known to maintain their stemness through the continuous induction of pluripotency transcription factors, such as Oct4, Nanog and Sox-2 [2,3,4,5].

There are several means of CSC identification including the determination of CSC-related surface markers, the detection of pluripotent transcription factors and investigation of CSC-like phenotypes. In lung cancer, several CSC markers have been identified and widely utilized, such as CD133, aldehyde dehydrogenase1A1 (ALDH1A1), Oct4 and Nanog [6,7]. In terms of clinical association, the presence of these CSC markers in the tumour tissue is related to poor clinical outcomes in lung cancer patients [8,9]. In addition, the expressions of Oct4, Nanog and other CSC marker proteins are correlated with high tumorigenicity and increased cancer aggressiveness [10,11].

As CSCs become dominant targets for novel anti-cancer therapy, the up-stream mechanism as well as the key determining signalling pathway that control stem cell properties have been intensively investigated. Among them, ATP-dependent tyrosine kinase (Akt), a central signal of cell survival and proliferation, has been linked with high CSC properties in a number of cancers. The enhanced Akt signal or the constantly activated Akt frequently observed in lung cancer are associated with the evasion of cell apoptosis, chemotherapeutic resistance and increased cell dissemination [12,13]. Importantly, the active Akt was demonstrated to enhance CSC properties and increase tumorigenicity through the upregulation of Oct4 and Nanog [14,15]. The discovery of new substances targeting CSC maintenance signals may improve the clinical outcomes in lung cancer patients and be used as an alternative therapy for disrupting CSC-driven lung cancer.

The plant-derived compound pongol methyl ether (PME) is a phenolic compound isolated from the stem bark of *Millettia erythrocalyx* Gagnep [16]. It was demonstrated in previous studies that the *Millettia erythrocalyx* Gagnep has several biologically active compounds [17], in particular, compounds possessing anti-cancer capability against several cancer types [18,19], however, the CSC-targeting as well as Akt inhibition are largely under investigated. Given that the effect of PME on CSC and Akt targeted approaches has not been revealed, this study aimed at determining the CSC-targeting activity of PME and its potential underlying mechanism of action blocking CSC-inductive pathways.

## 2. Results

### 2.1. Cytotoxicity and Anti-Proliferative Effect of PME on H460 Lung Cancer Cells

**Figure 1:**
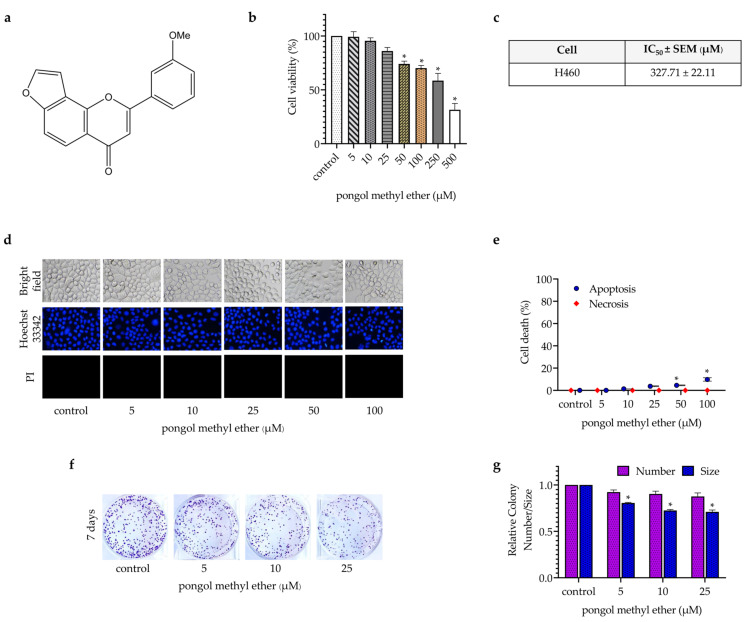
Cytotoxic effect of PME. (**a**) The chemical structure of PME. (**b**) H460 cells were treated with PME at various concentrations (0–500 μM) for 24 h, and cell viability was measured. (**c**) IC_50_ of H460 cells at 24 h of PME treatment. (**d**,**e**) Apoptotic and necrotic cell death were evaluated by Hoechst 33342/PI staining and calculated as a percentage compared with non-treated control cells. (**f**,**g**) Cells were treated with PME at non-toxic concentrations (0–25 µM), and colony was stained by crystal violet after 7 days. Data are presented as the mean ± SEM (*n* = 3). * *p* < 0.05 compared with nontreated cells.

To diminish the interference from the cytotoxic effect of PME on CSC phenotypes, we first evaluated the non-cytotoxic concentrations of PME on H460 cells. The cells were treated with various concentrations of PME (0–500 µM), and cell viability was determined after 24 h by 3-(4,5-dimethythiazol-2-yl)-2,5-diphenyl tetrazolium bromide assay. The results showed that PME has non-toxic concentrations below 50 µM (Figure 1b). Data analysis revealed that the IC_50_ of PME was approximately 327.71 μM (Figure 1c). Hoechst 33342 and propidium iodide nuclear staining assay indicated that the PME treatment at 0–25 µM had no significant effect on the apoptosis or necrosis of cells. The significant increase in apoptosis was observed when the cells treated with PME at 50 and 100 µM showed approximately 5% and 10% increase in apoptotic cell death, respectively (Figure 1d,e). We also confirmed the effect of PME on cell proliferation by colony formation assay, and the results showed that PME at 5–25 µM can significantly decrease the size of colonies (Figure 1f,g).

### 2.2. PME Attenuates CSC Phenotypes during Anchorage-Independent Growth and Spheroid Formation

**Figure 2:**
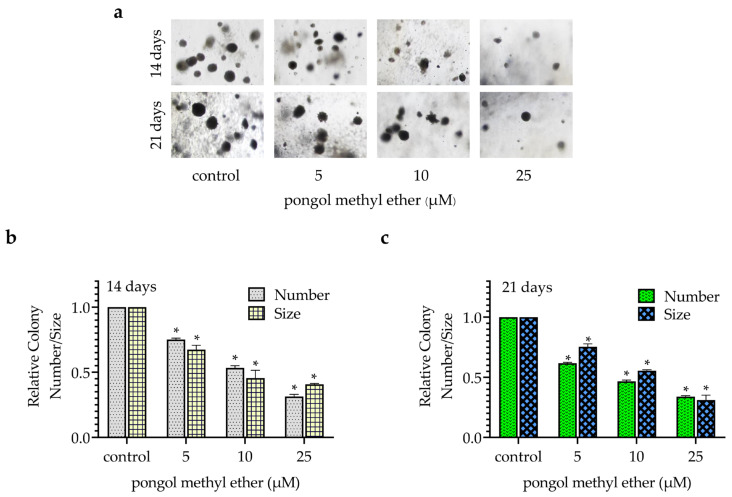
PME suppresses anchorage-independent growth. (**a**) H460 cells were pre-treated with PME (0–25 μΜ) for 24 h and subjected to an anchorage-independent growth assay. (**b**,**c**) Colony number and size were evaluated after day 14 and 21. Data are represented as mean ± SEM (*n* = 3) * *p* < 0.05 compared with untreated cells.

CSCs can form spheroids and grow in an anchorage-independent condition [4,20]. Cells were pre-treated with PME (0–25 µM) for 24 h, followed by examination of their anchorage-independent growth and spheroid formation. For anchorage-independent growth, the colony number and size were determined and are presented as relative values in comparison with those of non-treated control. Figure 2a reveals that PME significantly decreased the colony number and size in a concentration-dependent manner. A significant suppression of colony growth was first detected at 5 µM PME, with approximately 25 and 30% reduction in the colony number and size at 14 days, respectively (Figure 2b). Moreover, PME at 25 µM can reduce 65 and 70% of the colony number and size, respectively, at 21 days (Figure 2c).

**Figure 3:**
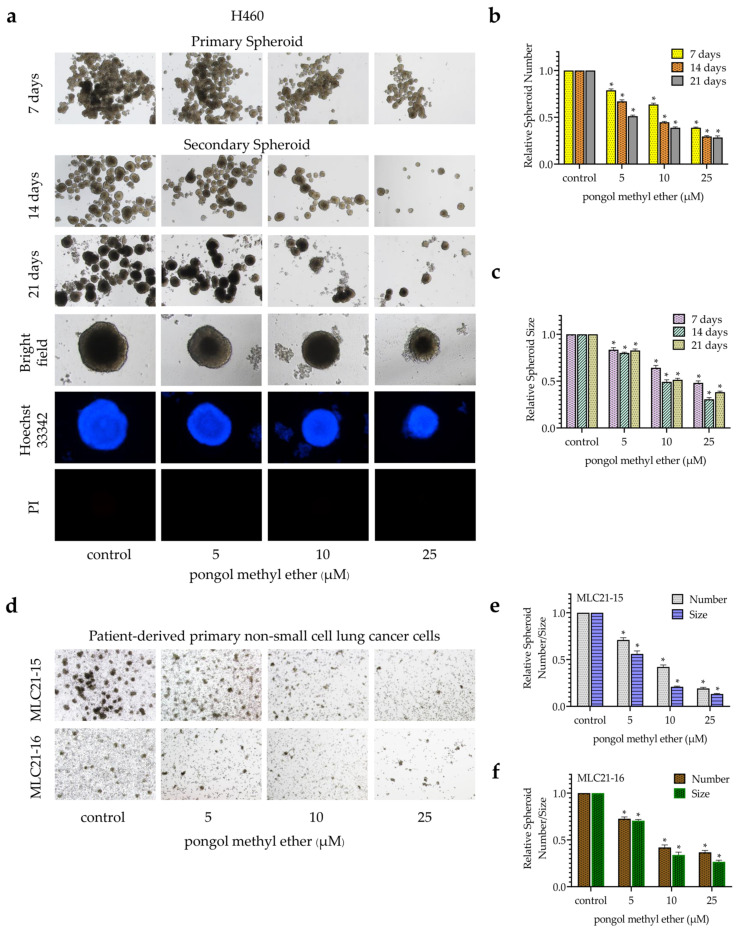
PME suppresses CSC-spheroid formation. (**a**) H460 cells were treated with PME (0–25 μΜ) for 24 h and subjected to spheroid formation assay. (**b**,**c**) The spheroid number and size were analysed and presented as the relative value to the control cells of each condition. (**d**) CSC-spheroids of patient-derived NSCLC cells were treated with PME (0–25 μΜ). (**e**,**f**) After 7 days, the spheroid number and size were determined. Data are represented as mean ± SEM (*n* = 3) * *p* < 0.05 compared with untreated cells.

For spheroid formation, the cells were pre-treated with PME (0–25 µM) for 24 h. Then, the cells were detached, re-suspended and seeded at a low density onto ultralow attachment plates. The non-treated cells formed spheroids, whereas the cells treated with PME exhibited a significant reduction in spheroids in a concentration-dependent manner (Figure 3a). Interestingly, the treatment of cells with PME at concentrations of 5–25 µM significantly reduced the number and size in the primary and secondary spheroids (Figure 3b,c), suggesting that PME at non-toxic concentrations can attenuate CSC phenotypes in terms of spheroid formation and anchorage-independent growth in H460 cells.

Having demonstrated the CSC-suppressing effects of this compound in human non-small cell lung cancer (NSCLC) cell line, we next investigated whether PME can attenuate the CSC phenotypes in patient-derived NSCLC cells. CSC spheroids of patient-derived NSCLC cells were treated with non-toxic concentrations of PME (0–25 µM). The results indicated that PME significantly reduced the ability of such cells to form spheroids compared with the control (Figure 3d–f).

### 2.3. PME Reduces CSC Markers and Pluripotent Transcription Factors through the Reduction of the Akt Signalling Pathway

**Figure 4:**
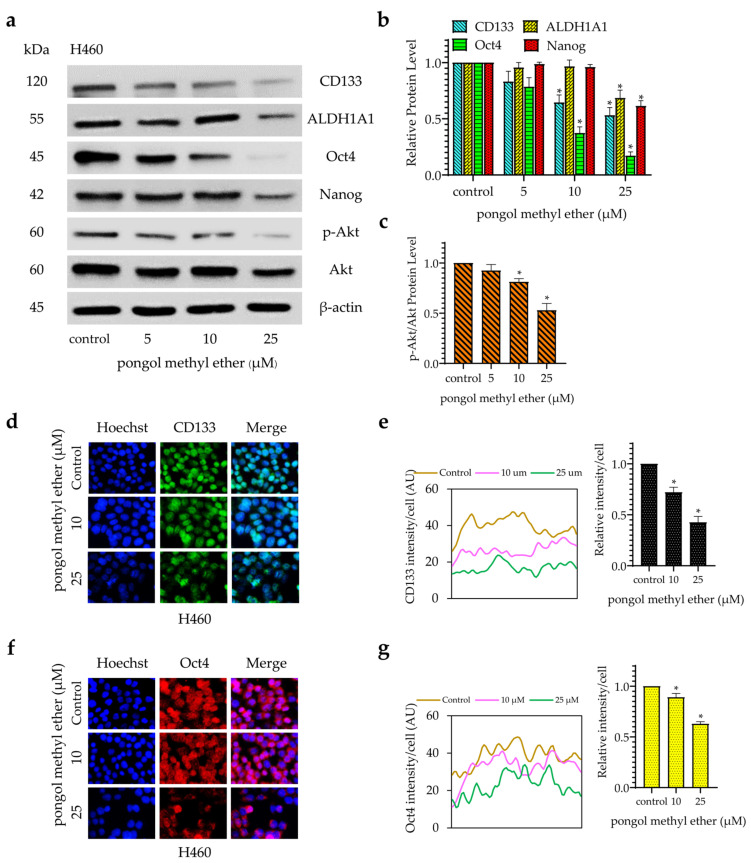
PME reduces CSC markers and transcription factors through inhibition of the ATP-dependent tyrosine kinase (Akt) signalling pathway. (**a**) H460 cells were treated with PME (0–25 μM) for 24 h, the levels of CD133, ALDH1A1, Oct4, Nanog, phosphorylated Akt and total Akt were examined by Western blotting. The blots were reprobed with antibody against β-actin. (**b**,**c**) Relative protein levels were quantified by densitometric analysis using ImageJ. (**d**) H460 cells were treated with PME (0, 10, 25 μM) for 24 h. The cells were co-stained with anti-CD133 antibodies and Hoechst 33342. (**e**) The expression of CD133 was examined using immunofluorescence. (**f**) The cells were co-stained with anti-Oct4 antibodies and Hoechst 33342. (**g**) The expression of Oct4 was examined using immunofluorescence. The fluorescence intensity was analysed by ImageJ software. Values are means ± SEM calculated as relative values to the non-treated control value. Data are represented as mean ± SEM (*n* = 3). * *p* < 0.05 compared with untreated cells.

**Figure 5:**
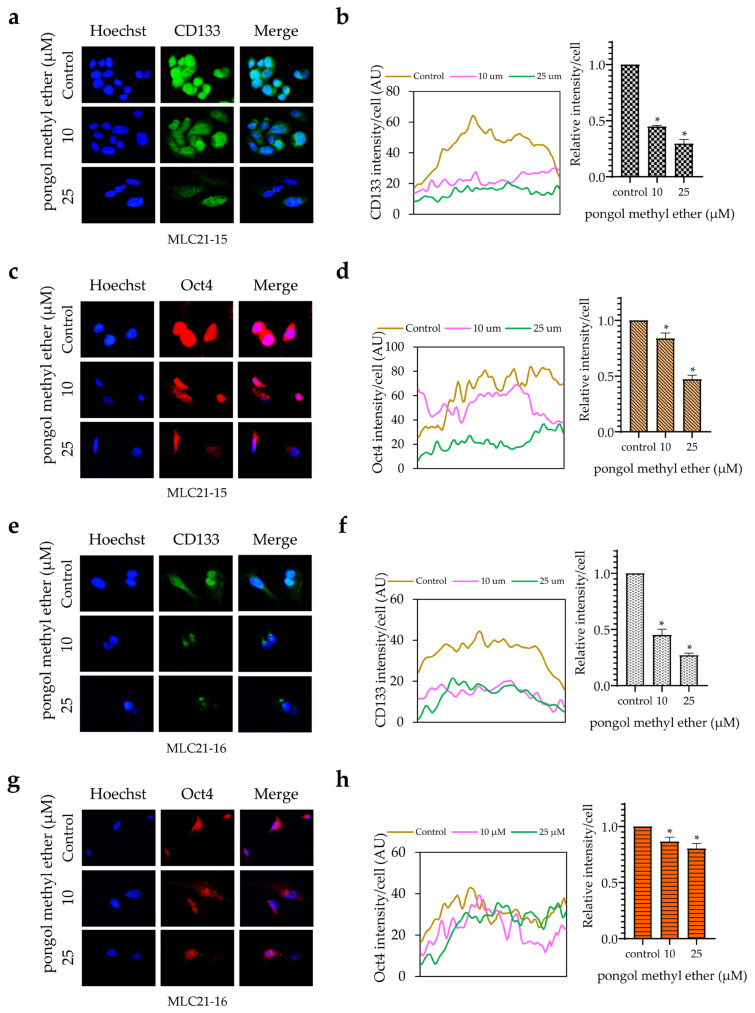
PME suppresses CSC marker (CD133) and transcription factor (Oct4) in patient-derived NSCLC cells. MLC21-15 and MLC21-16 were treated with non-toxic concentrations of PME (0, 10, 25 μM) for 24 h. The cells were co-stained with anti-CD133 antibodies (**a**,**e**); anti-Oct4 antibodies (**c**,**g**) and Hoechst 33342. The expression of CD133 (**b**,**f**) and Oct4 (**d**,**h**) were examined using immunofluorescence. The fluorescence intensity was analysed by ImageJ software. Values represent the mean ± SEM. (*n* = 3). * *p* < 0.05 compared with untreated cells.

Next, the expression of CSC-specific markers in the cells in response to PME were investigated. H460 cells were incubated with PME (0–25 µM) for 24 h, and the expression levels of CD133 and ALDH1A1 were determined by Western blot analysis. Figure 4a,b show that the treatment of cells with PME significantly reduced CD133 expression at 10 µM, whereas ALDH1A1 was reduced at 25 µM, respectively. The activated Akt controls the survival, growth and self-renewal abilities of CSCs [12]. In addition, Akt induces CSC phenotypes through the upregulation of Oct4 and Nanog [14,15]. The expression levels of phosphorylated Akt, total Akt, Oct4 and Nanog were evaluated by Western blotting. Figure 4a,c reveal that PME reduced the level of phosphorylated Akt. Similarly, the stem-cell transcription factors Oct4 and Nanog significantly decreased during PME treatment at concentrations 10 and 25 µM, respectively (Figure 4a,b). Altogether, our results suggest that PME suppressed the CSC phenotypes through Akt inhibition and depleted the stem-cell transcription factors. The PME depletion effects on CD133 and Oct4 were confirmed by immunofluorescence staining. Figure 4d–g show that PME significantly decreased the levels of CD133 and Oct4 in H460 cells. Persistently, similar results from the immunofluorescence analysis were found in patient-derived NSCLC cells Figure 5. These results showed that PME attenuated the CSC phenotypes and suppressed CSC makers and transcription factors via the inhibition of the Akt signalling pathway in human NSCLC cells.

### 2.4. Molecular Docking Simulations Indicated the PME Interaction with the Akt-1 Protein

**Figure 6:**
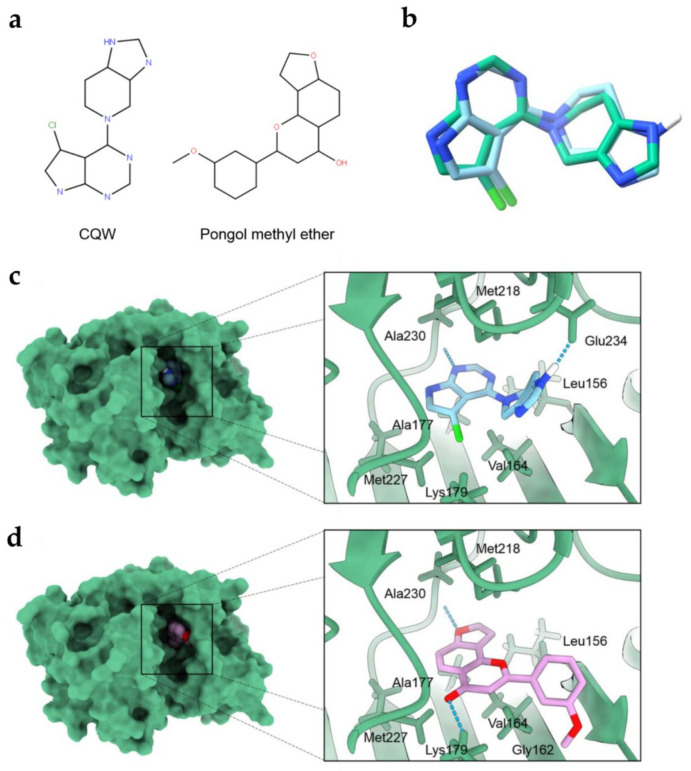
(**a**) The structure of CQW and PME. (**b**) Redocking of CQW in Akt-1 (PDB ID: 3CQW); CQW from the crystal (green) and CQW generated by redocking (blue). Docking interaction profile of Akt-1 inhibitors: (**c**) Akt-1 in complexed with CQW (reference compound), (**d**) Akt-1 in complexed with PME. The blue dashed lines represent hydrogen-bonding interaction.

To validate the docking protocol, we redocked CQW into its original binding site of Akt-1 (PDB ID: 3CQW). The root mean square deviation (RMSD) of the redocked ligand was 1.381 Å. The results (Figure 6b) indicate that the docking protocol was accurate (RMSD < 2 Å) [21].

In the molecular docking simulations, the binding free energy (kcal/mol) of all compounds was demonstrated (Table 1). PME exhibited a greater binding affinity than the Akt-1 inhibitor (reference compound) CQW (−8.3 kcal/mol), with an AutoDock Vina docking score of −9.2 kcal/mol. The docking results indicated that PME has a high binding affinity for Akt-1.

Figure 6c,d illustrate the interaction of all compounds. The docking result of PME revealed that it contributed to the hydrophobic interactions with Leu156, Gly162, Val164, Ala177, Met218 and Met227, and formed hydrogen bonds with Lys179 and Ala230.

These results suggest that PME interacted in the ATP-binding site of Akt-1, which is similar to the binding site of the reference compound.

### 2.5. Bioinformatic Analysis of the CSC Marker Proteins in PME Treatment

**Figure 7:**
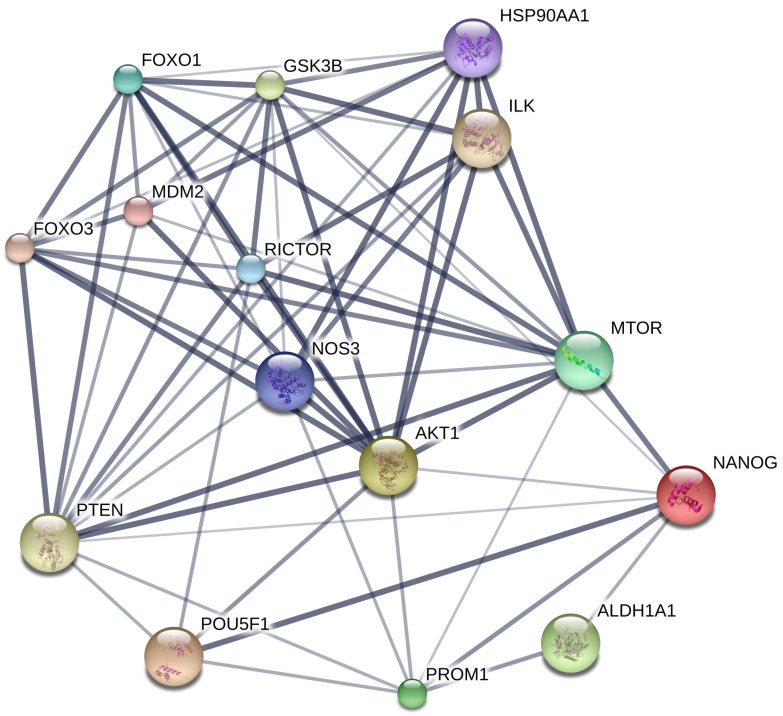
Protein-protein interaction (PPI) networks analysis of the CSC related proteins that affected by PME treatment: This is the confidence view; network nodes represent proteins, edges represent protein-protein associations, stronger associations are represented by thicker lines, protein-protein interactions are shown in grey, chemical-protein interactions in green and interactions between chemicals in red.

Having revealed the proteins involving in PME suppression of CSC in lung cancer cells, we next aimed at investigating the dominant protein form all detected protein using bioinformatic tool. The protein–protein interaction (PPI) networks and Kyoto Encyclopedia of Genes and Genomes (KEGG) pathway maps were used to construct the interaction of the CSC marker proteins in PME treatment. Search Tool for Interactions of Chemicals (STITCH) Version 5.0 (http://stitch.embl.de, accessed on 23 July 2021) was employed to identify the binding partners for NANOG, POU5F1 (POU class 5 homeobox 1; Oct4), AKT1, ALDH1A1 and PROM1 (prominin 1; CD133) and generate a protein interaction network. A total of 15 prominent protein nodes and 65 edges, including MTOR (mechanistic target of rapamycin), FOXO1 (forkhead box O1), RICTOR (RPTOR independent companion of MTOR), NOS3 (nitric oxide synthase 3), HSP90AA1 (heat shock protein 90 kDa alpha, class A member 1), MDM2 (mouse double minute 2), FOXO3 (forkhead box O3), ILK (integrin-linked kinase), PTEN (phosphatase and tensin) and GSK3β (glycogen synthase kinase 3 beta), were identified in this network (Figure 7). AKT1 was the most interactive protein in this PPI network and connected with 13 protein nodes. We identified AKT1 as a central protein in response to the PME treatment.

**Figure 8:**
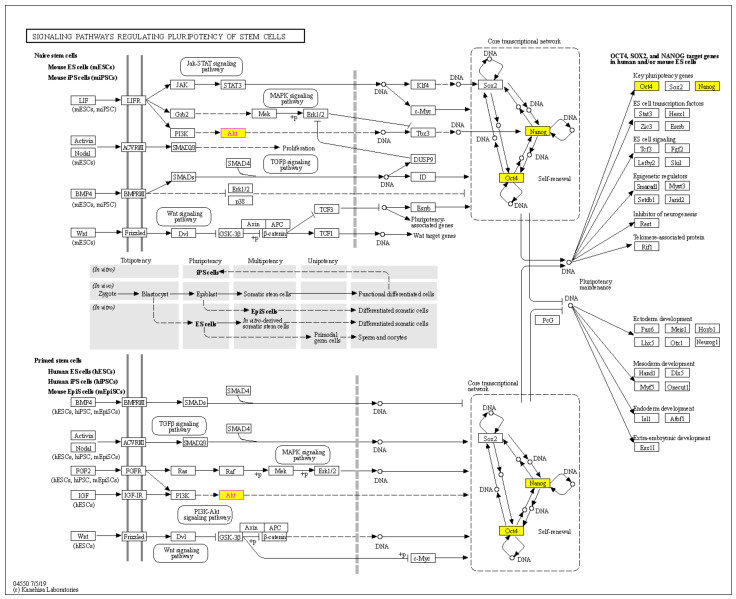
The KEGG mapper database revealed potential Akt up-stream and down-stream signals involved in pluripotency of stem cells. The yellow box represents the proteins affected by PME treatment.

To confirm the protein interaction and underlying pathway of PME treatment. The KEGG mapper (https://www.genome.jp/kegg/mapper.html, accessed on 23 July 2021) was utilised to construct the signalling pathway. The KEGG pathways related to the pluripotency of stem cell were identified, namely, ‘signalling pathways regulating pluripotency of stem cells’ (Figure 8). The KEGG pathway indicated that Akt was an important player in the mechanism of action of PME in the suppression of pluripotent transcription factors (Oct4 and Nanog) via the Akt inhibition. Moreover, Figure 8 also suggested other intermediate protein involving in Akt regulating stem cell transcription factors such as T-Box Transcription Factor 3 (Tbx3). In addition, this map suggested the possible up-stream regulator of Akt signals including the leukaemia inhibitory factor (LIF), fibroblast growth factor 2 (FGF2) and Insulin-like growth factor (IGF). Oct4 and Nanog were represented to be downstream target genes of such pathways that regulate self-renewal and pluripotency maintenance.

## 3. Discussion

Lung cancer is the most common cancer worldwide, in which normal epithelial cells undergo genetically damage induced cell proliferation without control in various parts of the lung, and these cancer cells can invade the surrounding tissues and metastasize to other distant organs [1,22]. CSCs are believed as the underlying cause of the high mortality rate in cancer [23]. Moreover, CSCs and related pathways have become potential targets of anti-cancer drugs. Previous research showed that phenolic compounds, such as vanillin, gigantol and lusianthridin, have the potential to suppress CSCs [24,25,26]. Gigantol, a bibenzyl compound from *Dendrobium draconis*, suppresses CSC phenotypes in lung cancer cells [25]. Vanillin, a major component in *Vanilla planifolia* seed, reduces CSC phenotypes in lung cancer cells and downregulates CSC markers CD133, ALDH1A1 and ATP-binding cassette super-family G member 2 (ABCG2) [24]. In addition, lusianthridin, a dihydrophenanthrene compound isolated from the stem of *Dendrobium venustum*, can suppress lung CSC phenotypes and decrease the levels of CD133, ALDH1A1 and ABCG2 [26].

In this study, we demonstrated for the first time that PME attenuated the CSC phenotypes in human lung cancer cells. The treatment of these cancer cells with PME resulted in their decreased anchorage-independent growth and spheroid formation (Figure 2 and Figure 3). To determine the CSCs, we used CSC markers CD133 and ALDH1A1, which are widely used in the case of lung cancer [6,7]. CSCs were related to the ability of cells to establish survive colonies in an anchorage-independent condition and form detached tumour spheroids [4,20]. We evaluated the expression of such CSC markers in PME-treated cells and observed that PME treatment can reduce CSC markers in concomitant with the suppression of tumour spheroid formation and limited growth in detached condition (Figure 4).

The regulation of CSC properties, such as self-renewal and pluripotency, is modulated by stem-cell transcription factors of normal stem cells and CSCs [3]. Oct4 and Nanog are transcription factors that indicate CSC properties in various types of cancers including lung cancer [3,7]. The expressions of Oct4 and Nanog induce spheroid and colony formation [7,20]. Moreover, their expression is related with lung cancer aggressiveness and increases new tumour genesis [27,28]. In this study, Oct4 and Nanog significantly decreased in response to the treatment with PME at non-toxic concentrations (Figure 4).

In terms of upstream signalling pathway, Akt is a cellular signalling pathway that plays a key role in regulating CSC abilities [12]. The regulation of CSC transcription factors, including Oct4 and Nanog, showed the downstream regulation of the Akt signalling pathway, causing tumorigenicity and cancer aggressiveness [12,13,14,15]. The inhibition of Akt activity attenuated the activity of these transcription factors and other CSC marker proteins, which diminished the resulting CSC phenotypes [12,29]. Previous studies described the potential anti-CSC benefit of CSC transcription factors/Akt pathway inhibition. Srinual et al. reported that vanillin suppresses Oct4 and Nanog expression through the mediation by an Akt-dependent mechanism [24]. Gigantol suppresses Oct4 and Nanog reduction through an Akt-dependent mechanism [25]. Moreover, both of studies confirm this mechanism using perifosine (1,1-dimethylpiperidinium-4-yl octadecyl phosphate), an Akt inhibitor as a positive control. They treated H460 cells with non-toxic concentrations of perifosine and detect the CSC markers by Western blot analysis. The result revealed that treatment of H460 cells with perifosine significant reduced p-Akt and specific CSCs markers [24,25]. In our findings, PME inhibited CSCs through the reduction of transcription factors Oct4 and Nanog in an Akt-dependent mechanism (Figure 4). To confirm our hypothesis, we performed molecular docking simulations using the ATP-binding site of Akt-1 protein as the target for this compound and investigated Akt-1 interactions compared with the Akt-1 inhibitor (reference compound; CQW). After simulations (Figure 6), the data indicated that the binding affinity of PME was greater than that of the reference compound and had a similar binding site, which demonstrated the capability of PME to inhibit Akt-1, following previous experimental results.

To further confirm the underlying pathway of PME treatment, we constructed the PPI networks and KEGG pathway maps. To investigate the PPI networks and identify the key protein target of this treatment, using STITCH database, we analysed the CSC marker proteins that were affected by the PME treatment. We observed that AKT1 was the top protein with the central PPI degree, indicating that PME may exert its CSC-suppressing activity through an Akt-dependent mechanism (Figure 7). In the KEGG pathway map analysis, we recorded that the CSC marker proteins that were affected by the PME treatment were mainly enriched in the signalling pathways regulating the pluripotency of stem cells (Figure 8). This pathway revealed that Akt is an upstream regulator of pluripotent transcription factors Oct4 and Nanog, following the previous experimental results on PME treatment. In addition, this pathway also revealed that LIF, FGF2 and IGF could be upstream regulators of PI3K-Akt signalling pathway. Previous studies indicated that such receptors have an ability to regulate CSCs [30,31,32,33,34,35]. LIF receptor (LIFR), an upstream regulator of Hippo signalling, has an inversely expressed in relation with miR-125a in human breast cancer stem cells [30]. FGF receptor (FGFR), one of the most common growth factors, regulates renewal and differentiation of human embryonic stem cells (hESCs) and CSCs [31,32]. In addition, the IGF signalling was shown to induce and maintain CSC and epithelial mesenchymal transition (EMT) status [33]. Shan et al. reported that Nanog regulates self-renewal of CSCs through the IGF pathway in human hepatocellular carcinoma [34]. The inhibition of IGF receptors (IGFR)/Akt/MTOR axis targets colorectal CSCs by attenuating mevalonate-isoprenoid pathway in vitro and in vivo [35]. Moreover, this KEGG mapper revealed that Oct4 and Nanog were key pluripotent genes of such pathways to regulate self-renewal and pluripotency maintenance. Most of these proteins that affected by the PME treatment have been reported in lung CSCs, confirming the reliability of the results of bioinformatic analysis.

Moreover, we confirmed the CSC suppression of the compound in primary lung cells derived from advanced stage and recurrent NSCLC patients who had been treated with chemotherapeutic drugs for a prolonged period, which supports the potential use of PME in lung cancer therapy. The results of immunofluorescence analysis revealed that PME can reduce CSC-specific markers expression (CD133 and Oct4) and suppress spheroid formation (Figure 3 and Figure 5). Similar with a previous phenolic compound study, it is reported that lusianthridin could suppress CSCs and reduced the ability to form spheroids in primary lung cancer cells [26].

## 4. Materials and Methods

### 4.1. Isolation of Pongol Methyl Ether

PME, 2-(3-Methoxyphenyl)-4H-furo[2,3-h]-1-benzopyran-4-one, was isolated from the stem bark of *Millettia erythrocalyx* Gagnep. as previously indicated [16]. A chemical formula of PME is shown in Figure 1a.

### 4.2. Cell Lines and Cultures

The human NSCLC cell lines H460 was obtained from the American Type Culture Collection (Manassas, VA, USA). For primary lung cancer cells, the protocol was approved by the Ethics Committee of the Faculty of Medicine, Chulalongkorn University, Bangkok, Thailand (IRB 365/62) (Date of Approval: 31 July 2020). The malignant pleural effusion was collected from a part of standard diagnosis practice and clinical treatment by thoracentesis. All cells were cultured in Roswell Park Memorial Institute (RPMI) 1640 medium (Gibco, Grand Island, NY, USA) at 37 °C with 5% CO_2_.

### 4.3. Reagents and Antibodies

RPMI 1640 medium, L-glutamine and trypsin-EDTA were obtained from Gibco (Grand Island, NY, USA). 3-(4, 5-dimethylthiazol-2-yl)-2,5-diphenyltetrazolium bromide (MTT), Propidium iodide (PI) and Hoechst 33342 were purchased from Sigma-Aldrich, Co. (St. Louis, MO, USA). Antibodies for CD133 (#CA1217) were obtained from Cell Applications, Inc. (San Diego, CA, USA), while ALDH1A1(#36671), Oct4 (#2750), Nanog (#4903), Akt (#9272), phosphorylated Akt (#4060) and β-actin (#4970), as well as peroxidase conjugated secondary antibodies were obtained from Cell Signaling Technology, Inc. (Danvers, MA, USA).

### 4.4. Cell Viability Assay

After treatment, the RPMI media was removed and replaced with MTT solution, incubated at 37 °C for 4 h. Then the MTT solution was removed and 100 µL DMSO added to dissolve the formazan crystal, and the absorbance was measured at 570 nm using a microplate reader (Anthros, Durham, NC, USA). The cell viability was calculated as follows:

% Cell viability = (OD of treatment group/OD of control group) × 100(1)

### 4.5. Nuclear Staining Assay

After treatment, cells were incubated with Hoechst 33342 (Sigma, St. Louis, MO, USA) and propidium iodide (PI) (Sigma, St. Louis, MO, USA), 10 μM at 37 °C for 30 min. Cells were visualized and imaged under a fluorescence microscopy (Olympus DP70, Melville, NY, USA). Apoptotic and necrotic cell death were scored and analysed as the percentages of all cells viewed.

### 4.6. Colony Formation Assay

Cells were pre-treated with PME at non-toxic concentrations (0–25 μM and incubated for 24 h at 37 °C before subjected to the assay. Culture RPMI media (200 μL/well) was fed to the system every 3 days. Then the colony was stained with 0.1% crystal violet for 30 min at room temperature and rinsed with PBS. Colony formation was assessed and the results were counted from three independent experiments (*n* = 3). Colony size and number were determined using ImageJ 1.52 v software (http://imagej.nih.gov/ij/index.html, Bethesda, MD, USA/accessed on 3 December 2020) compared with the control group.

### 4.7. Anchorage-Independent Growth Assay

To prepare the lower layer, a combination of RPMI media and melted 1% agarose (Bio-Rad, Hercules, CA, USA) was used at a 1:1 ratio, then 500 μL of this mixture was put in a 24-well plate and allowed to solidify at 4 °C for 20 min. To prepare the upper layer, melted 0.3% agarose and RPMI media with 10% FBS (Merck, Darmstadt, Germany) containing pre-treated H460 cells at a density of 1 × 10^3^ cells/mL was used, then 250 μL of this mixture was added as an upper layer. After the upper layer was solidified, the cultured RPMI media was added over the upper layer and incubated at 37 °C for 3 weeks. Further cultured RPMI media (200 μL/well) was applied every 3 days to prevent the soft agar drying. Colony formation was determined after 3 weeks using a phase-contrast microscope (Nikon ECLIPSE Ts2, Tokyo, Japan). Colony number and size were counted and determined using ImageJ 1.52 v software compared with the control group.

### 4.8. Spheroid Formation Assay

Pre-treated cells were cultured in a 24-well ultra-low attachment plate at a density of 5 × 10^3^ cells/mL in serum-free RPMI media and incubated at 37 °C for 7 days. Primary spheroids formed and were photographed at day 7 by using a phase-contrast microscope (Nikon ECLIPSE Ts2, Tokyo, Japan). Then primary spheroids were resuspended into a single cell and 5 × 10^3^ cells/mL were cultured onto a 24-well ultralow attachment plates for 14 days. Secondary spheroids were allowed to form and were photographed at day 14 and day 21. The spheroid number and size were analysed compared with the control group.

### 4.9. Western Blot Analysis

After treatment, the cell lysates were collected, and protein content was measured by using BCA protein assay kit from Pierce Biotechnology (Rockford, IL, USA). Protein samples (40 μg) were separated onto 10% sodium dodecyl sulphate polyacrylamide gel electrophoresis (SDS-PAGE) and transferred to polyvinylidene difluoride (PVDF) (Bio-Rad Laboratories Inc., Hercules, CA, USA). Membranes were blocked for 30 min with 5% nonfat-milk (Merck, DA, Germany) and incubated with the primary antibody at 4 °C overnight. The membranes were washed and incubated with secondary antibody at room temperature for 2 h and detected by chemiluminescent solution (Supersignal West Pico;Pierce, Rockford, IL, USA) and the proteins expression levels were quantified by ImageJ 1.52v software.

### 4.10. Immunofluorescence Assay

Cells were fixed with 4% paraformaldehyde for 20 min and permeabilized with 0.1% Triton-x in PBS for 20 min. Then, cells were blocked, washed and incubated with primary antibody overnight at 4 °C, washed, and incubated with secondary antibodies. Cells were incubated with Hoechst 33342 (Sigma, St. Louis, MO, USA) and visualized by fluorescence microscopy with a 40× objective lens (Nikon ECLIPSE Ts2, Tokyo, Japan).

### 4.11. Protein and Ligands Preparation

The protein structure of the Akt-1 complexed with the ligand inhibitor (CQW) was downloaded from the Research Collaboratory for Structural Bioinformatics Protein Data Bank (RCSB PDB) [36] at 2 Å (PDB ID: 3CQW) [37]. Before docking simulation, all water molecules and ligand were removed with the UCSF ChimeraX [38]. Hydrogen atoms added with the program reduce [39] in AutoDockFR [40]. The 3D structure of CQW was extracted from 3CQW PDB code and used as a reference. The 3D structure of PME was download from the PubChem database [41] (Pubchem CID: 636768). The gaussian 09 program [42] applied to optimize the geometry of the ligands using density functional theory (DFT) with a B3LYP/6-31G (d,p) basis set. These ligand structures are illustrated in Figure 6a.

### 4.12. Molecular Docking

In the molecular docking simulation, AutoDock vina [43] was employed to clarify both binding mode and affinity of the selected potential Akt-1 inhibitor. A grid box was set with the centre of the co-crystallized ligand inhibitor (CQW of 3CQW) and dimension (x = 20, y = 20, and z = 20), with a spacing of 1 Å [44]. The exhaustiveness parameter was set to 24 [45]. Other AutoDock vina parameters were set as default. The binding pattern of protein–ligand interaction was analysed using the UCSF ChimeraX.

### 4.13. Bioinformatics Analysis; PPI Networks Integration and KEGG Pathway Maps Analyses

To identify the interaction between the CSC marker proteins that affected by PME treatment, the PPI networks were construct using STITCH (http://stitch.embl.de/; version 5.0/accessed on 23 July 2021) online database. After that, the CSC marker proteins were also encoded to the network-based classification of KEGG Ontology (KO) as follows: Akt (K04456), Oct4 (K09367), Nanog (K10164), CD133 (K06532) and ALDH1A1 (K07249) before analysis. KEGG pathway maps analysis and functional annotation for the selected encoded proteins were performed by utilizing involved signalling pathways associated with CSCs (https://www.genome.jp/kegg, accessed on 23 July 2021).

### 4.14. Statistical Analysis

The data from three independent experiments (*n* = 3) are presented as the mean ± standard error of the mean (S.E.M.) Statistical differences between multiple groups were analyzed using an analysis of variance (ANOVA) and post-hoc test at a significance level of *p* < 0.05 (*).

## 5. Conclusions

**Figure 9:**
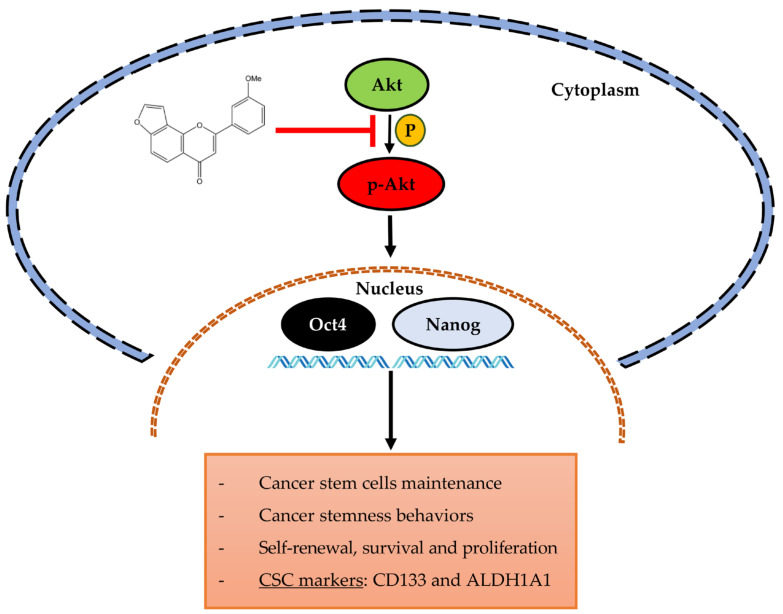
The schematic representation the attenuation effect of PME on CSC phenotypes and its underlying mechanism pathway in human NSCLC cells.

In conclusion, this study reported the novel information of PME in the suppression of CSC phenotypes in human NSCLC cells via the inhibition of Akt signalling. The Akt inhibitory effect resulted in the suppression of stem-cell transcription factors and the depletion of CSCs (Figure 9). These data can support the potential use of PME in lung cancer therapy.

## Figures and Tables

**Table 1:** Binding free energy of the docking simulations (kcal/mol).

No.	Compounds	Free Energy of Binding (kcal/mol)
1	Pongol methyl ether	−9.2
2	(CQW) reference compound	−8.3

## Data Availability

Data is contained within the article.
